# Medical Items

**Published:** 1894-01

**Authors:** 


### *MEDICAL ITEMS.*

The advertising firm of Hummel & Parmele, Philadelphia, has dissolved. Dr. A. S. Hummel continues the business.

The Columbia Chemical Co. (formerly The Health Restorative Co.), of No. 90 S. 5th Ave., New York, has removed to No. 1704 G St., N. W., Washington, D. C.

OUR esteemed quacks, Amick Consumption Cure Company, have sued the editor of the Cincinnati *Lancet-Clinic* for $100,000. We hope the suit will result like the one against Dr. Reeves, of Chattanooga.

SUCH confusion of names has existed among our Pacific coast exchanges that one of them, the *Pacific Medical Record* (Portland, Oregon), has changed its name to the *Medical Sentinel.* The editorial and business management remain the same.

PROFESSOR JOHN TYNDALL, the eminent English scientist, died from an overdose of chloral hydrate December 4th. Prof. Tyndall had for some time been taking a dose of the syrup of chloral at night and a dose of magnesium sulphate solution in the morning. On the fatal day seven drams of the chloral solution were given him in place of the magnesium, and he died in a few hours.

SIR ANDREW CLARK, Physician-Extraordinary to the Queen and to Honorable Mr. Gladstone, died in November. Dr. Clark's medical cereer was a most brilliant one and crowned with the highest honors. At the time of his death he occupied the Chair of Clinical Medicine in the London hospital. Associated with Mr. Jonathan Hutchinson and others, he had for many years published the lectures and reports of the Medical Staff of the London hospital. He was buried in Westminster Abbey with imposing ceremonies. Mr. Gladstone was one of the pall-bearers.

Professor Nicholas Senn, of Chicago, has given his medical library to the Newberry Library. This library was one of the most valuable and magnificent collections of medical works in the world. The greater part of it was bought by Professor Senn a few years ago from the estate of Professor William Baum, Professor of Surgery in the University of Gottingen. When sent to this country the collection of books, pamphlets, etc., constituted an entire carload. The library is composed mainly of works on surgery, gynecology, ophthalmology, Virchow's and Longenbeck's Archives, etc. Such a generous gift will not fail to be appreciated by the public and profession of Chicago.

---

CITY SUBSCRIBERS.—You will confer a favor by notifying us of any failure of our carrier to deliver your JOURNAL.

